# Selection

**Published:** 1900-03

**Authors:** 


### SELECTION.

*Farmers' Bulletin*, No. 101, issued by the Department of Agriculture, relative to value of millet as a food, has this to say as to the reputed injuriousness of foxtail millet forage:

In some sections of the country the foxtail millets have gained the reputation of being injurious to certain kinds of stock, and are therefore regarded with suspicion by many farmers and stockmen. Like many other forage-plants, these millets become very harsh and woody with age, and are then difficult of thorough mastication and hard to digest. Then, too, at this stage of growth the beards are stiff and harsh, and are not only difficult to digest, but produce more or less irritation in the digestive tract of the animal, and sometimes unite with other indigestible substances, forming compact balls in the stomach, ultimately causing death. This difficulty may be avoided by cutting the hay in proper season, as recommended elsewhere. No more trouble seems to have been experienced in feeding the millets in the fresh state than with any other succulent forage. Most of the injury has arisen from feeding the hay in large quantities with little or no other grain or forage, and for extended periods.

At the North Dakota experiment station an extended experiment was recently conducted to determine what, if any, deleterious effects would result to horses from a continued diet in which millet hay replaced that ordinarily used in the ration. The animals were grained, watered, and otherwise cared for as usual. At the end of the experiment Dr. Hinebauch,[1] the veterinarian of the station, concluded that millet, when used alone as a coarse food, is injurious to horses—first, in producing an increased action of the kidneys; second, in causing lameness and swelling of the joints; third, in producing infusion of blood into the joints; fourth, in destroying the texture of the bone, rendering it softer and less tenacious, so that traction causes the ligaments and muscles to be torn loose.

These results seem to show conclusively that under certain conditions millet-hay becomes injurious to horses at least, if not to other stock also. This agrees, too, with the general experience of farmers and stockmen, that a long-continued diet of millet-hay, particularly hay in poor condition, tends to weaken horses and unfit them for doing hard work. Again, both immature and overripe foxtail millet is said to produce abortion in brood-mares and cows, but this has not been established experimentally.

More recently Professor Ladd,[2] of the same experiment station, has made a chemical study of millet-hay, in which he succeeded in isolating a glucoside which he calls setarian, and which, from its physiological action on mice, rats, and young cats, he concludes to be the cause of the injury to horses and other stock.

Millet in any stage of growth acts as a laxative and diuretic. At times the action is more pronounced than at others. Thus hay cut while the plants are quite young seems to be most strongly laxative, while overripe hay is most strongly diuretic. However, if the hay is cut at the right stage of growth and properly cured, the action in either case will not be sufficient to lead to serious results if other hay or coarse forage is fed along with the millet. One feed of millet-hay per day for work-horses, and one or two for other stock, is sufficient, and when fed in this manner the millet acts as a stimulant and alterative, and tends to produce and maintain a healthy condition of the animals.

1 North Dakota Experiment Station Bul. No. 26, p. 105, 1896.

2 North Dakota Experiment Station Bul, No. 35, p. 323, 1899. See also Amer. Chem. Journal, No. 20, p. 861.

